# RETRACTION: Extracts of *Eucalyptus alba* Promote Diabetic Wound Healing by Inhibiting *α*‐Glucosidase and Stimulating Cell Proliferation

**DOI:** 10.1155/ecam/9838064

**Published:** 2026-04-28

**Authors:** Evidence-Based Complementary and Alternative Medicine

RETRACTION: R. Mumtaz, M. Zubair, M. A. Khan, S. Muzammil, and M. H. Siddique, “Extracts of *Eucalyptus alba* Promote Diabetic Wound Healing by Inhibiting *α*‐Glucosidase and Stimulating Cell Proliferation,” *Evidence-Based Complementary and Alternative Medicine*, 2022, 4953105, https://doi.org/10.1155/2022/4953105.

The above article, published online on 15 April 2022 in Wiley Online Library (https://wileyonlinelibrary.com), has been retracted by John Wiley & Sons Ltd. The retraction has been agreed due to errors identified in Figures 5 and 8, initially raised on PubPeer [1]. On further investigation, a summary of the concerns is as below:

In Figure 8, there are:

• Overlapping regions between panels g and l
• Overlapping regions between panels b and c
• Overlapping regions between panels d and i
• Overlapping regions between panels a and b
• Overlapping regions between panels a and c
• Overlapping regions between panels i and j
• Overlapping regions between panels g and h
• Overlapping regions between panels d and j
• Overlapping regions between panels g and m
• Overlapping regions between panels h and l
• Panel l shares unexpected, repeated areas with panel m in Fig 4 of [2].
• Panel e shares unexpected, repeated areas with panel g in Fig 4 of [2].

In Figure 5,

• Panel h shares unexpected, repeated areas with panel i in Fig 6 of [2]
• Panel l shares unexpected, repeated areas with panel d in Fig 6 of [2].

The authors provided a response; however, after assessment, the results are deemed unreliable, and the article is retracted.

The authors disagree with the retraction.
